# Neurologic Manifestations of Pandemic (H1N1) 2009 Virus Infection

**DOI:** 10.3201/eid1603.091699

**Published:** 2010-03

**Authors:** Sarawut Kitcharoen, Moragot Pattapongsin, Kittisak Sawanyawisuth, Vincent Angela, Somsak Tiamkao

**Affiliations:** Khon Kaen University, Khon Kaen, Thailand (S. Kitcharoen, K. Sawanyawisuth, S. Tiamkao); Chaiyaphum Hospital, Chaiyaphum, Thailand (M. Pattapongsin); University of Oxford, Oxford, UK (V. Angela)

**Keywords:** influenza A pandemic (H1N1) 2009 virus, pandemic (H1N1) 2009, influenza, viral hemorrhagic fevers, occupational exposure, curriculum, viruses, letter

**To the Editor:** In April 2009, the outbreak of influenza A pandemic (H1N1) 2009 virus was reported. Subsequently, the disease spread throughout the world, and the pandemic alert level was raised to level 6 in June by the World Health Organization. Pandemic (H1N1) 2009 virus infection spread to Thailand and is now found throughout Thailand. Similar to the effects of other viruses, pandemic (H1N1) 2009 virus may cause neurologic complications. Associated neurologic symptoms were first reported from Dallas, Texas, USA: 4 children experienced unexplained seizures or had an alteration of consciousness level that was associated with this virus (*1*). We report an adult patient with pandemic (H1N1) 2009 infection who had neurologic complications.

A 34-year-old man, previously healthy, was admitted to Chaiyaphum Hospital in Chaiyaphum, Thailand, on August 24, 2009, with influenza-like symptoms. Two days after admission, progressive quadriparesis with bilateral, symmetric paresthesia (glove-and-stocking pattern), and areflexia developed. His motor weakness (grades III/V) began in both legs and then involved both arms and hands. Other neurologic examinations showed limitation of extraocular movement in all directions, normal pupil size and light reflex, and facial diplegia. A lumbar puncture was performed, and cerebrospinal fluid (CSF) contained neither leukocytes nor erythrocytes, with a protein level of 19.5 mg/dL.

On day 3 after the patient’s admission, acute respiratory failure developed. A nasopharyngeal aspirate specimen was positive for pandemic (H1N1) 2009 virus by PCR. The patient received oseltamivir, zanamivir, and ventilator support. His chest radiograph showed diffuse alveolar infiltration. On day 10, his motor weakness worsened to grade 0, and his consciousness level was diminished to a drowsy state.

A computed tomography scan of the brain showed diffuse white matter lesions (Figure). Repeated lumbar punctures continued to show CSF findings within the reference range. An electrophysiologic study, electromyogram, and nerve conduction study showed polyneuropathy, axonopathy type. Guillain-Barré syndrome was suspected, and intravenous immunoglobulin was given for 5 days. Tests for GQ1b and GM1 antibodies were carried out at Oxford University; results were negative.

**Figure:**
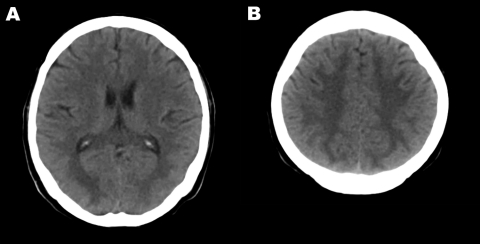
Computed tomography images of the brain of an adult patient with pandemic (H1N1) 2009 virus infection and neurologic signs. A noncontrast study showed hypodense lesions in both occipital lobes (A) and in both upper parietal lobes (B).

Other laboratory tests showed mild transaminitis and negative results for syphilis testing and for serologic tests for HIV, hepatitis B virus, hepatitis C virus, Japanese encephalitis virus, herpes simplex virus, and *Mycoplasma pneumoniae*. A CSF antigen test was negative, and CSF culture was negative for bacteria. Meropenem was given to treat ventilator-associated pneumonia, which was caused by β-lactam–resistant *Klebsiella pneumoniae*. After a month of treatment, the patient regained consciousness, his motor strength improved considerably, and he was able to be gradually removed from the ventilator. After 3 months, he was discharged with self-assisted status.

Our report shows neurologic manifestations associated with pandemic (H1N1) 2009 virus infection in an adult. The manifestation of progressive quadriplegia with diffuse sensory loss is compatible with a polyneuropathy. The neurologic signs developed 2 days after the respiratory tract signs.

Although a diagnosis of Guillain-Barré syndrome was considered initially, according to the National Institute of Neurologic Disorders and Stroke criteria (*2*), some clinical features did not support this diagnosis. These included the lack of CSF albuminocytologic dissociation, the fact that the clinical signs occurred during the outbreak of pandemic (H1N1) 2009 virus infection rather than after it, and the fact that antibodies were not found in gangliosides. CSF albuminocytologic dissociation and serum ganglioside antibodies may be found in 85%–90% of Guillain-Barré syndrome patients (*2*).

Alternatively, the patient might have had central nervous system complication from pandemic (H1N1) 2009 virus infection. Acute disseminated encephalomyelitis is a condition that might occur within 30 days after an infectious process (*3*). It can lead to quadriplegia and diffuse white matter lesions. The clinical feature that makes acute disseminated encephalomyelitis less likely in this patient was the CSF findings in the reference range. In summary, however, we believe that pandemic (H1N1) 2009 virus infection can cause neurologic complications affecting both the peripheral and central nervous systems in adult patients.
